# 1,2-Bis(pyridin-4-yl)ethene–4-hy­droxy-3-meth­oxy­benzoic acid (1/1)

**DOI:** 10.1107/S2414314622003042

**Published:** 2022-03-29

**Authors:** Devin J. Angevine, Jason B. Benedict

**Affiliations:** a Department of Chemistry, The State University of New York at Buffalo, Buffalo, New York 14260-3000, USA; University of Aberdeen, Scotland

**Keywords:** crystal structure, organic co-crystal, vanillic acid, bi­pyridine ethyl­ene

## Abstract

In the title 1:1 co-crystal [alternatively called bi­pyridine ethyl­ene–*p*-vanillic acid (1/1)], C_12_H_10_N_2_·C_8_H_8_O_4_, the dihedral angle between the pyridine rings is 59.51 (5)°. In the crystal, the mol­ecules are linked by O—H⋯N hydrogen bonds, generating [401] chains of alternating C_12_H_10_N_2_ and C_8_H_8_O_4_ mol­ecules.

## Abstract 2

A co-crystal consisting of a 1:1 ratio of *p*-vanillic acid and bi­pyridine ethyl­ene was synthesized. A series of O—H⋯N inter­actions connect the mol­ecules into twisting wires, which are cross-linked through van der Waals inter­actions.

## Graphical abstract

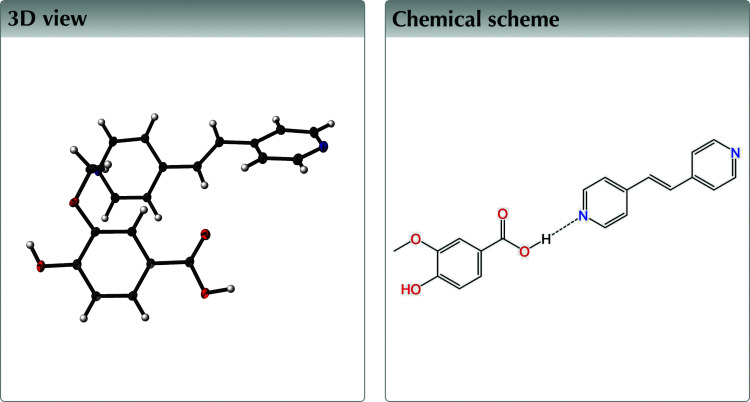

## Structure description

4-Hy­droxy-3-meth­oxy­benzoic acid, C_8_H_8_O_4_, known commonly as *p*-vanillic acid, is used as a flavoring agent and naturally found in a variety of fruits and edible plants (Ingole *et al.*, 2021 ▸). In addition, *p*-vanillic acid is currently being investigated for its inflammatory pain-inhibiting properties (Calixto-Campos *et al.*, 2015 ▸). Despite the prevalence of the mol­ecule in our foods and its potential medicinal benefits, structural information on vanillic acid is sparse with few crystal structures being reported thus far. As such it is crucial to expand the number of structures containing vanillic acid in order to better understand the non-covalent inter­actions involving this mol­ecule. Bi­pyridine ethyl­ene (C_12_H_10_N_2_; BPyE) was selected as a suitable coformer for the present study because of its ability to form both simple and complex hydrogen-bonded networks with organic acids (Delori *et al.*, 2013 ▸; Bhattacharya *et al.*, 2013 ▸).

**Figure 1:**
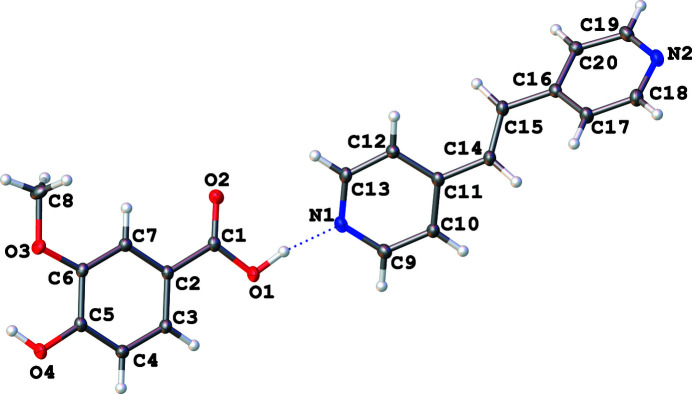
A bimolecular unit consisting of *p*-vanillic acid and BPyE with the hydrogen bond depicted as a blue dashed line. The BPyE mol­ecule illustrated is generated by the symmetry operation *x* – 1, *y*, *z* from the asymmetric mol­ecule.

**Figure 2:**
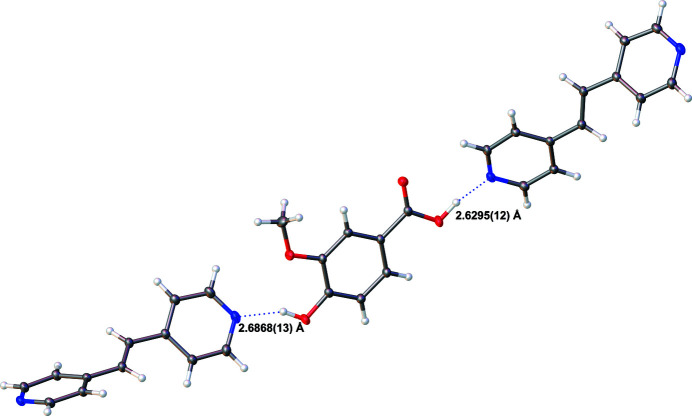
Part of a [401] hydrogen-bonded chain of *p*-vanillic acid and BPyE mol­ecules. The O⋯N distances are shown for each O—H⋯N hydrogen-bonding inter­action.

**Figure 3:**
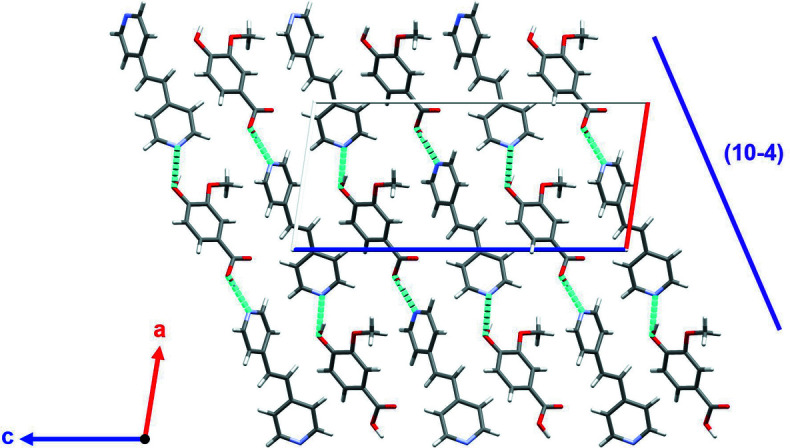
plane depicting twisting hydrogen-bonded wires running approximately parallel to (10 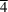 ). Hydrogen-bonding inter­actions are depicted as bright-blue dashed lines.

When *p*-vanillic acid is combined with BPyE in a 1:1 molar ratio, the resulting 1:1 co-crystal possesses monoclinic (*P*2_1_/*c*) symmetry at 90 K. The vanillic acid has two distinct O—H⋯N-type hydrogen-bonding inter­actions (Table 1 ▸); one of these involves the carb­oxy­lic acid group and a BPyE N atom acceptor and resulting in a 2.6295 (12) Å distance between heteroatoms (Fig. 1 ▸). The other hydrogen bond occurs between the *para*-position hydroxyl group and the other pyridine N atom of a BPyE mol­ecule resulting in a 2.6868 (13) Å distance between heteroatoms (Fig. 2 ▸). The co-crystal structure may be described as dimolecular units made up of one acid plus one coformer, which form 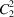 (19) chain motifs. These chains propagate in the [401] direction, forming twisting wires (Fig. 3 ▸). The wires stack along [010], forming sheets, which subsequently form layers parallel to (10 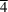 ), with every other sheet being rotated 180° about [010]. Two weak C—H⋯O contacts are also observed (Table 1 ▸).

## Synthesis and crystallization

A 1:1 molar ratio of bi­pyridine ethyl­ene (182.2 mg, 1 mmol) and *p*-vanillic acid (168.1 mg, 1 mmol) was added to a 25 ml scintillation vial to which methanol was added until both compounds dissolved (approximately 20 ml). The resulting solution was vortexed for 30 s at 3000 rpm on a VWR Mini Vortexer MV I. The solution was then stored in the dark uncapped to allow for crystal formation while the solvent slowly evaporated.

## Refinement

Crystal data, data collection, and structure refinement details are summarized in Table 2 ▸.

## Supplementary Material

Crystal structure: contains datablock(s) I. DOI: 10.1107/S2414314622003042/hb4402sup1.cif

Structure factors: contains datablock(s) I. DOI: 10.1107/S2414314622003042/hb4402Isup2.hkl

Supporting information file. DOI: 10.1107/S2414314622003042/hb4402Isup3.cml

CCDC reference: 2160226

Additional supporting information:  crystallographic information; 3D view; checkCIF report

## Figures and Tables

**Table 1:** Hydrogen-bond geometry (Å, °)

*D*—H⋯*A*	*D*—H	H⋯*A*	*D*⋯*A*	*D*—H⋯*A*
O1—H1⋯N1^i^	0.99 (2)	1.65 (2)	2.6295 (12)	169 (2)
O4—H4⋯N2^ii^	0.92 (2)	1.84 (2)	2.6868 (13)	154 (2)
C4—H4*A*⋯O2^iii^	0.95	2.53	3.2341 (14)	132
C9—H9⋯O3^iv^	0.95	2.45	3.3520 (14)	158

Symmetry codes: (i) 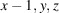 ; (ii) 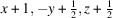 ; (iii) 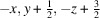 ; (iv) 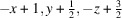 .

**Table 2:** Experimental details

Crystal data	
Chemical formula	C_12_H_10_N_2_·C_8_H_8_O_4_
*M*_r_	350.36
Crystal system, space group	Monoclinic, *P*2_1_/*c*
Temperature (K)	90
*a*, *b*, *c* (Å)	9.1486 (5), 9.2114 (5), 20.3429 (12)
β (°)	98.416 (1)
*V* (Å^3^)	1695.86 (16)
*Z*	4
Radiation type	Mo *K*α
μ (mm^−1^)	0.10
Crystal size (mm)	0.54 × 0.22 × 0.02

Data collection	
Diffractometer	Bruker APEXII CCD
Absorption correction	Multi-scan (*SADABS*; Bruker, 2016 ▸)
*T*_min_, *T*_max_	0.648, 0.746
No. of measured, independent and observed [*I* > 2σ(*I*)] reflections	33598, 5958, 4683
*R*_int_	0.084
(sin θ/λ)_max_ (Å^−1^)	0.748

Refinement	
*R*[*F* ^2^ > 2σ(*F* ^2^)], *wR*(*F* ^2^), *S*	0.047, 0.131, 1.03
No. of reflections	5958
No. of parameters	245
H-atom treatment	H atoms treated by a mixture of independent and constrained refinement
Δρ_max_, Δρ_min_ (e Å^−3^)	0.40, −0.26

Computer programs: *APEX2* and *SAINT* (Bruker, 2016 ▸), *SHELXT2018/2* (Sheldrick, 2015*a* ▸), *SHELXL2018/3* (Sheldrick 2015*b* ▸), and *OLEX2* (Dolomanov *et al.*, 2009 ▸).

## full crystallographic data

### Crystal data

**Table:**

C_12_H_10_N_2_·C_8_H_8_O_4_	*F*(000) = 736
*M**_r_* = 350.36	*D*_x_ = 1.372 Mg m^−^^3^
Monoclinic, *P*2_1_/*c*	Mo *K*α radiation, λ = 0.71073 Å
*a* = 9.1486 (5) Å	Cell parameters from 5974 reflections
*b* = 9.2114 (5) Å	θ = 2.4–32.1°
*c* = 20.3429 (12) Å	µ = 0.10 mm^−^^1^
β = 98.416 (1)°	*T* = 90 K
*V* = 1695.86 (16) Å^3^	Plate, clear colourless
*Z* = 4	0.54 × 0.22 × 0.02 mm

### Data collection

**Table:**

Bruker APEXII CCD diffractometer	4683 reflections with *I* > 2σ(*I*)
φ and ω scans	*R*_int_ = 0.084
Absorption correction: multi-scan (SADABS; Bruker, 2016)	θ_max_ = 32.1°, θ_min_ = 2.0°
*T*_min_ = 0.648, *T*_max_ = 0.746	*h* = −13→13
33598 measured reflections	*k* = −13→13
5958 independent reflections	*l* = −30→30

### Refinement

**Table:**

Refinement on *F*^2^	Hydrogen site location: mixed
Least-squares matrix: full	H atoms treated by a mixture of independent and constrained refinement
*R*[*F*^2^ > 2σ(*F*^2^)] = 0.047	*w* = 1/[σ^2^(*F*_o_^2^) + (0.0409*P*)^2^ + 0.6347*P*] where *P* = (*F*_o_^2^ + 2*F*_c_^2^)/3
*wR*(*F*^2^) = 0.131	(Δ/σ)_max_ = 0.001
*S* = 1.03	Δρ_max_ = 0.40 e Å^−^^3^
5958 reflections	Δρ_min_ = −0.26 e Å^−^^3^
245 parameters	Extinction correction: *SHELXL2018/3* (Sheldrick 2015b), Fc^*^=kFc[1+0.001xFc^2^λ^3^/sin(2θ)]^-1/4^
0 restraints	Extinction coefficient: 0.0070 (15)
Primary atom site location: dual	

### Special details

**Table:**

Geometry. All esds (except the esd in the dihedral angle between two l.s. planes) are estimated using the full covariance matrix. The cell esds are taken into account individually in the estimation of esds in distances, angles and torsion angles; correlations between esds in cell parameters are only used when they are defined by crystal symmetry. An approximate (isotropic) treatment of cell esds is used for estimating esds involving l.s. planes.
Refinement. The O-bound H atoms were located in difference maps and their positions were freely refined. The C-bound H atoms were placed geometrically (C—H = 0.95–0.98 Å) and refined as riding atoms with *U*_iso_(H) = 1.2*U*_eq_(C) or 1.5*U*_eq_(methyl C).

### Fractional atomic coordinates and isotropic or equivalent isotropic displacement parameters (Å^2^)

**Table:**

	*x*	*y*	*z*	*U*_iso_*/*U*_eq_	
O3	0.43471 (9)	0.32423 (9)	0.77939 (4)	0.01865 (17)	
O2	−0.05986 (9)	0.39976 (10)	0.62580 (4)	0.01877 (17)	
O4	0.41561 (9)	0.50304 (10)	0.88365 (4)	0.02072 (18)	
O1	−0.17538 (9)	0.55862 (10)	0.68391 (4)	0.02055 (18)	
N1	0.58124 (10)	0.49305 (11)	0.60383 (5)	0.01647 (18)	
N2	−0.30499 (10)	0.09310 (11)	0.39212 (5)	0.0198 (2)	
C11	0.30840 (11)	0.38151 (12)	0.54449 (5)	0.01433 (19)	
C5	0.30531 (11)	0.49848 (12)	0.83165 (5)	0.01447 (19)	
C1	−0.06039 (11)	0.47842 (12)	0.67408 (5)	0.01454 (19)	
C2	0.06755 (11)	0.49121 (12)	0.72818 (5)	0.01337 (19)	
C3	0.06593 (11)	0.58633 (12)	0.78115 (5)	0.01466 (19)	
H3	−0.016259	0.648781	0.782394	0.018*	
C7	0.19036 (11)	0.40137 (12)	0.72604 (5)	0.01356 (19)	
H7	0.192506	0.337576	0.689504	0.016*	
C16	−0.01655 (11)	0.18988 (12)	0.43775 (5)	0.01485 (19)	
C12	0.44140 (11)	0.31630 (12)	0.53464 (5)	0.0158 (2)	
H12	0.441106	0.232395	0.507463	0.019*	
C6	0.30870 (11)	0.40521 (12)	0.77703 (5)	0.01375 (19)	
C9	0.45404 (12)	0.55722 (13)	0.61287 (5)	0.0171 (2)	
H9	0.458134	0.641952	0.639702	0.021*	
C10	0.31694 (11)	0.50520 (12)	0.58473 (5)	0.0154 (2)	
H10	0.229348	0.553233	0.592721	0.019*	
C13	0.57369 (11)	0.37518 (13)	0.56488 (5)	0.0164 (2)	
H13	0.663253	0.329869	0.557653	0.020*	
C15	0.13307 (11)	0.24385 (13)	0.46164 (5)	0.0163 (2)	
H15	0.210971	0.220751	0.437269	0.020*	
C4	0.18456 (11)	0.58996 (12)	0.83222 (5)	0.0158 (2)	
H4A	0.183227	0.655853	0.868048	0.019*	
C20	−0.06769 (12)	0.17485 (13)	0.36998 (5)	0.0172 (2)	
H20	−0.004731	0.196381	0.338053	0.021*	
C14	0.16281 (11)	0.32450 (13)	0.51668 (5)	0.0163 (2)	
H14	0.082548	0.346774	0.539750	0.020*	
C17	−0.11344 (12)	0.15200 (13)	0.48206 (6)	0.0180 (2)	
H17	−0.083003	0.159193	0.528642	0.022*	
C18	−0.25442 (12)	0.10380 (13)	0.45737 (6)	0.0192 (2)	
H18	−0.318447	0.077004	0.488118	0.023*	
C19	−0.21164 (12)	0.12807 (13)	0.34974 (6)	0.0199 (2)	
H19	−0.245544	0.120512	0.303447	0.024*	
C8	0.44161 (13)	0.22430 (14)	0.72620 (6)	0.0221 (2)	
H8A	0.430464	0.277176	0.683957	0.033*	
H8B	0.361902	0.152884	0.725170	0.033*	
H8C	0.537185	0.174302	0.732989	0.033*	
H4	0.499 (2)	0.458 (3)	0.8743 (11)	0.055 (6)*	
H1	−0.259 (3)	0.528 (3)	0.6504 (12)	0.066 (7)*	

### Atomic displacement parameters (Å^2^)

**Table:**

	*U*^11^	*U*^22^	*U*^33^	*U*^12^	*U*^13^	*U*^23^
O3	0.0149 (3)	0.0218 (4)	0.0183 (4)	0.0073 (3)	−0.0006 (3)	−0.0023 (3)
O2	0.0165 (4)	0.0236 (4)	0.0156 (4)	0.0004 (3)	0.0004 (3)	−0.0028 (3)
O4	0.0136 (4)	0.0285 (5)	0.0181 (4)	0.0034 (3)	−0.0043 (3)	−0.0043 (3)
O1	0.0116 (3)	0.0257 (4)	0.0226 (4)	0.0033 (3)	−0.0031 (3)	−0.0063 (3)
N1	0.0135 (4)	0.0191 (5)	0.0156 (4)	−0.0005 (3)	−0.0016 (3)	0.0012 (3)
N2	0.0141 (4)	0.0209 (5)	0.0229 (5)	−0.0007 (3)	−0.0020 (3)	−0.0022 (4)
C11	0.0129 (4)	0.0171 (5)	0.0125 (4)	0.0002 (4)	0.0003 (3)	0.0012 (4)
C5	0.0123 (4)	0.0158 (5)	0.0148 (4)	−0.0010 (3)	0.0006 (3)	0.0010 (4)
C1	0.0119 (4)	0.0158 (5)	0.0157 (4)	−0.0006 (3)	0.0012 (3)	0.0016 (4)
C2	0.0107 (4)	0.0147 (5)	0.0144 (4)	−0.0004 (3)	0.0005 (3)	0.0009 (3)
C3	0.0118 (4)	0.0152 (5)	0.0167 (4)	0.0017 (3)	0.0010 (3)	−0.0003 (4)
C7	0.0131 (4)	0.0142 (5)	0.0134 (4)	−0.0001 (3)	0.0021 (3)	0.0002 (3)
C16	0.0125 (4)	0.0154 (5)	0.0159 (4)	0.0013 (3)	−0.0004 (3)	−0.0015 (4)
C12	0.0138 (4)	0.0172 (5)	0.0156 (4)	0.0015 (4)	−0.0004 (3)	−0.0020 (4)
C6	0.0115 (4)	0.0142 (5)	0.0154 (4)	0.0018 (3)	0.0015 (3)	0.0016 (4)
C9	0.0164 (5)	0.0181 (5)	0.0158 (5)	0.0005 (4)	−0.0010 (4)	−0.0012 (4)
C10	0.0126 (4)	0.0189 (5)	0.0145 (4)	0.0015 (4)	0.0008 (3)	−0.0011 (4)
C13	0.0120 (4)	0.0199 (5)	0.0166 (5)	0.0021 (4)	0.0000 (3)	0.0009 (4)
C15	0.0119 (4)	0.0201 (5)	0.0164 (5)	0.0002 (4)	0.0008 (3)	0.0002 (4)
C4	0.0135 (4)	0.0176 (5)	0.0160 (4)	0.0002 (4)	0.0015 (3)	−0.0030 (4)
C20	0.0161 (5)	0.0187 (5)	0.0164 (5)	−0.0001 (4)	0.0010 (4)	−0.0027 (4)
C14	0.0117 (4)	0.0197 (5)	0.0169 (5)	0.0002 (4)	0.0008 (3)	−0.0007 (4)
C17	0.0146 (4)	0.0222 (5)	0.0165 (5)	−0.0008 (4)	0.0001 (4)	0.0001 (4)
C18	0.0135 (5)	0.0221 (5)	0.0218 (5)	−0.0008 (4)	0.0018 (4)	0.0000 (4)
C19	0.0174 (5)	0.0226 (6)	0.0180 (5)	−0.0005 (4)	−0.0029 (4)	−0.0035 (4)
C8	0.0230 (5)	0.0235 (6)	0.0198 (5)	0.0101 (4)	0.0031 (4)	−0.0025 (4)

### Geometric parameters (Å, º)

**Table:**

O3—C6	1.3680 (12)	C16—C15	1.4705 (15)
O3—C8	1.4292 (14)	C16—C20	1.3963 (15)
O2—C1	1.2211 (13)	C16—C17	1.3979 (15)
O4—C5	1.3516 (12)	C12—H12	0.9500
O4—H4	0.92 (2)	C12—C13	1.3854 (15)
O1—C1	1.3243 (13)	C9—H9	0.9500
O1—H1	0.99 (2)	C9—C10	1.3858 (15)
N1—C9	1.3417 (14)	C10—H10	0.9500
N1—C13	1.3400 (15)	C13—H13	0.9500
N2—C18	1.3439 (15)	C15—H15	0.9500
N2—C19	1.3386 (16)	C15—C14	1.3379 (15)
C11—C12	1.3975 (15)	C4—H4A	0.9500
C11—C10	1.3984 (15)	C20—H20	0.9500
C11—C14	1.4661 (14)	C20—C19	1.3895 (15)
C5—C6	1.4083 (15)	C14—H14	0.9500
C5—C4	1.3908 (15)	C17—H17	0.9500
C1—C2	1.4892 (14)	C17—C18	1.3869 (15)
C2—C3	1.3906 (15)	C18—H18	0.9500
C2—C7	1.4012 (14)	C19—H19	0.9500
C3—H3	0.9500	C8—H8A	0.9800
C3—C4	1.3886 (14)	C8—H8B	0.9800
C7—H7	0.9500	C8—H8C	0.9800
C7—C6	1.3858 (14)		
			
C6—O3—C8	117.07 (9)	C10—C9—H9	118.6
C5—O4—H4	112.0 (14)	C11—C10—H10	120.2
C1—O1—H1	107.0 (14)	C9—C10—C11	119.55 (10)
C13—N1—C9	117.86 (9)	C9—C10—H10	120.2
C19—N2—C18	117.30 (10)	N1—C13—C12	123.09 (10)
C12—C11—C10	117.32 (10)	N1—C13—H13	118.5
C12—C11—C14	123.47 (10)	C12—C13—H13	118.5
C10—C11—C14	119.18 (9)	C16—C15—H15	119.0
O4—C5—C6	122.43 (9)	C14—C15—C16	121.99 (10)
O4—C5—C4	118.53 (10)	C14—C15—H15	119.0
C4—C5—C6	119.04 (9)	C5—C4—H4A	119.5
O2—C1—O1	123.31 (10)	C3—C4—C5	120.93 (10)
O2—C1—C2	123.10 (10)	C3—C4—H4A	119.5
O1—C1—C2	113.58 (9)	C16—C20—H20	120.4
C3—C2—C1	121.74 (9)	C19—C20—C16	119.30 (10)
C3—C2—C7	119.71 (9)	C19—C20—H20	120.4
C7—C2—C1	118.52 (9)	C11—C14—H14	117.1
C2—C3—H3	120.0	C15—C14—C11	125.72 (10)
C4—C3—C2	119.94 (10)	C15—C14—H14	117.1
C4—C3—H3	120.0	C16—C17—H17	120.3
C2—C7—H7	119.9	C18—C17—C16	119.35 (10)
C6—C7—C2	120.26 (10)	C18—C17—H17	120.3
C6—C7—H7	119.9	N2—C18—C17	123.28 (11)
C20—C16—C15	121.40 (10)	N2—C18—H18	118.4
C20—C16—C17	117.35 (10)	C17—C18—H18	118.4
C17—C16—C15	121.24 (10)	N2—C19—C20	123.38 (10)
C11—C12—H12	120.3	N2—C19—H19	118.3
C13—C12—C11	119.36 (10)	C20—C19—H19	118.3
C13—C12—H12	120.3	O3—C8—H8A	109.5
O3—C6—C5	114.87 (9)	O3—C8—H8B	109.5
O3—C6—C7	125.05 (10)	O3—C8—H8C	109.5
C7—C6—C5	120.07 (9)	H8A—C8—H8B	109.5
N1—C9—H9	118.6	H8A—C8—H8C	109.5
N1—C9—C10	122.81 (10)	H8B—C8—H8C	109.5
			
O2—C1—C2—C3	177.25 (11)	C12—C11—C14—C15	−26.50 (18)
O2—C1—C2—C7	−4.63 (16)	C6—C5—C4—C3	2.43 (16)
O4—C5—C6—O3	−2.61 (15)	C9—N1—C13—C12	−0.93 (16)
O4—C5—C6—C7	177.83 (10)	C10—C11—C12—C13	0.39 (16)
O4—C5—C4—C3	−177.84 (10)	C10—C11—C14—C15	155.42 (11)
O1—C1—C2—C3	−3.78 (15)	C13—N1—C9—C10	1.19 (16)
O1—C1—C2—C7	174.34 (10)	C15—C16—C20—C19	−177.84 (11)
N1—C9—C10—C11	−0.66 (17)	C15—C16—C17—C18	178.96 (11)
C11—C12—C13—N1	0.15 (17)	C4—C5—C6—O3	177.11 (9)
C1—C2—C3—C4	176.90 (10)	C4—C5—C6—C7	−2.45 (16)
C1—C2—C7—C6	−177.00 (9)	C20—C16—C15—C14	146.38 (12)
C2—C3—C4—C5	−0.61 (17)	C20—C16—C17—C18	−0.85 (17)
C2—C7—C6—O3	−178.84 (10)	C14—C11—C12—C13	−177.72 (10)
C2—C7—C6—C5	0.68 (16)	C14—C11—C10—C9	178.05 (10)
C3—C2—C7—C6	1.15 (16)	C17—C16—C15—C14	−33.42 (17)
C7—C2—C3—C4	−1.20 (16)	C17—C16—C20—C19	1.97 (17)
C16—C15—C14—C11	179.32 (10)	C18—N2—C19—C20	−0.37 (18)
C16—C20—C19—N2	−1.42 (19)	C19—N2—C18—C17	1.59 (18)
C16—C17—C18—N2	−0.98 (19)	C8—O3—C6—C5	177.99 (10)
C12—C11—C10—C9	−0.15 (16)	C8—O3—C6—C7	−2.47 (16)

### Hydrogen-bond geometry (Å, º)

**Table:**

*D*—H···*A*	*D*—H	H···*A*	*D*···*A*	*D*—H···*A*
O1—H1···N1^i^	0.99 (2)	1.65 (2)	2.6295 (12)	169 (2)
O4—H4···N2^ii^	0.92 (2)	1.84 (2)	2.6868 (13)	154 (2)
C4—H4*A*···O2^iii^	0.95	2.53	3.2341 (14)	132
C9—H9···O3^iv^	0.95	2.45	3.3520 (14)	158

Symmetry codes: (i) *x*−1, *y*, *z*; (ii) *x*+1, −*y*+1/2, *z*+1/2; (iii) −*x*, *y*+1/2, −*z*+3/2; (iv) −*x*+1, *y*+1/2, −*z*+3/2.

## References

[bb1] Bhattacharya, S., Stojaković, J., Saha, B. K. & MacGillivray, L. R. (2013). *Org. Lett.* **15**, 744–747. 10.1021/ol303283s 23350882

[bb2] Bruker (2016). *APEX2*, *SAINT* and *SADABS*. Bruker AXS Inc., Madison, Wisconsin, USA.

[bb3] Calixto-Campos, C., Carvalho, T. T., Hohmann, M. S. N., Pinho-Ribeiro, F. A., Fattori, V., Manchope, M. F., Zarpelon, A. C., Baracat, M. M., Georgetti, S. R., Casagrande, R. & Verri, W. A. (2015). *J. Nat. Prod.* **78**, 1799–1808. 10.1021/acs.jnatprod.5b00246 26192250

[bb4] Delori, A., Eddleston, M. D. & Jones, W. (2013). *CrystEngComm*, **15**, 73–77.

[bb5] Dolomanov, O. V., Bourhis, L. J., Gildea, R. J., Howard, J. A. K. & Puschmann, H. (2009). *J. Appl. Cryst.* **42**, 339–341.

[bb6] Ingole, A., Kadam, M., Dalu, A., Kute, S., Mange, P., Theng, V., Lahane, R., Nikas, A., Kawal, Y., Nagrik, S. & Patil, P. (2021). *J. Drug. Deliv. Ther.* **11**, 200–204.

[bb7] Sheldrick, G. M. (2015*a*). *Acta Cryst.* C**71**, 3–8.

[bb8] Sheldrick, G. M. (2015*b*). *Acta Cryst.* C**71**, 3–8.

